# Leaving no one behind: lessons from implementation of policies for universal HIV treatment to universal health coverage

**DOI:** 10.1186/s12992-020-00549-4

**Published:** 2020-02-24

**Authors:** Yibeltal Assefa, Peter S. Hill, Wim Van Damme, Judith Dean, Charles F. Gilks

**Affiliations:** 1 School of Public Health, the University of Queensland, Brisbane, Australia; 2 Institute of Tropical Medicine, Antwerp, Belgium

**Keywords:** Universal health coverage, Universality, Equity, Leaving no one behind, HIV, antiretroviral treatment

## Abstract

**Background:**

The third Sustainable Development Goal (SDG − 3) aims to ensure healthy lives and promote well-being for all at all ages. SDG-3 has a specific target on universal health coverage (UHC), which emphasizes the importance of all people and communities having access to quality health services without risking financial hardship. The objective of this study is to review progress towards UHC using antiretroviral treatment (ART) as a case study.

**Methods:**

We used a mixed-methods design including qualitative and quantitative approaches. We reviewed and synthesised the evidence on the evolution of the WHO HIV treatment guidelines between 2002 and 2019. We calculated ART coverage over time by gender, age group, and location. We also estimated ART coverage differences and ratios.

**Findings:**

ART guidelines have evolved from “treating the sickest” to “treating all”. ART coverage increased globally from under 7% in 2005 to 62% in 2018. There have been successes in increasing ART coverage in all populations and locations. However, progress varies by population and location in many regions. There is inequity in ART coverage: women (68%) versus men (55%), and adults (62%) versus children (54%). This inequity has widened over time, and with expanded ART eligibility criteria. On the other hand, data from at least one high-burden country (Ethiopia) shows that inequity among regions has narrowed over time due to the improvements in the primary health care systems and implementation of the public health approach in the country.

**Conclusion:**

ART coverage has increased at global, regional and national levels to all population groups. However, the gains have not been equitable among locations and populations. Policies towards universality may widen the inequity in resource-limited settings unless countries take precautions and “put the last first”. We argue that primary health care and public health approaches, with multi-sectoral actions and community engagement, are vital to minimize inequity, achieve UHC and leave no one behind.

## Background

Good health is essential to human welfare and to sustained economic and social development [1]. Initiatives to improve the health status of people are not only the right thing to do, but also pragmatic approaches to human development [2]. Timely access to health services, a mix of promotion, prevention, diagnosis, treatment and rehabilitation, is critical to maintain health and improve disease outcomes. Cognizant of this, in 2005, Member States of the World Health Organization (WHO) committed to universal health coverage (UHC) so that all, irrespective of background, have access to the health services they need without suffering financial hardship [3]. In December 2012, a resolution was passed by the United Nations General Assembly that recognized the centrality of UHC to enhancing health, social cohesion and sustainable human and economic development [4]. The 2030 agenda for sustainable development includes health as a central component of development: the third Sustainable Development Goal (SDG-3) aims to ensure healthy lives and promote well-being for all at all ages. SDG-3 has a specific target on UHC (target 3.8), which emphasizes the importance of all people and communities having access to quality health services without risking financial hardship [5].

Monitoring progress towards target 3.8 is crucial to improve implementation of the required activities for UHC. It focuses on two aspects: the proportion of a population that can access essential quality health services and the proportion of the population that spends a large amount of household income on health. WHO has identified 16 essential health services in four categories as indicators for monitoring progress towards UHC: (1) Reproductive, maternal, newborn and child health: family planning, antenatal and delivery care, full child immunization, and health-seeking behaviour for pneumonia; (2) infectious diseases: tuberculosis treatment, HIV antiretroviral treatment (ART), Hepatitis treatment, use of insecticide-treated bed nets for malaria prevention, and adequate sanitation; (3) Non-communicable diseases: prevention and treatment of raised blood pressure, prevention and treatment of raised blood glucose, cervical cancer screening, and tobacco (non-)smoking; and (4) Service capacity and access: basic hospital access, health worker density, access to essential medicines, and health security [6].

Antiretroviral treatment for HIV is one of the targets used to monitor progress towards UHC albeit from a vertical disease control perspective. HIV programs have been implementing strategies towards universal access to services long before the commitment for UHC. Countries have been scaling up ART programs for more than two decades, with major scientific advances and extensive implementation experience over this period [7]. These advances have been accompanied by significant improvements in antiretroviral medicines (price, dosage, toxicity) that have enabled major changes in the HIV treatment guidelines [8].

There have been successes and challenges in the implementation of these WHO guidelines (towards UHC for HIV services). We argue that we can learn from the experience in scaling up HIV services, as a case study for achieving UHC. The objective of this paper is to review the progress using HIV treatment, which is one of the indicators used by WHO to monitor UHC, as an example. The evidence from this study will assist the global response in moving forward to achieving universal coverage for ART, in particular, and the health SDG, in general.

## Methods

We reviewed and synthesised the evidence on the evolution of the WHO ART guidelines between 2002 and 2019, and analysed data on the number of people on ART, the number of new HIV infections and deaths among PLHIV. We also reviewed changes in ART coverage over time by gender, age group, and location. This study is based on data from the Joint United Nations Programme on HIV/AIDS (UNAIDS) data base (which compiled data reported from countries every year) [9], and studies from Ethiopia, which has a high HIV burden, the second largest population in Africa, and a federal structure [10]. HIV testing coverage, number of people on ART, ART coverage, new HIV infections and AIDS-related deaths were the key variables we extracted and analysed the data for this study.

We calculated ART coverage differences and ART coverage ratios (*parity index*) comparing female with male, children with adults and provinces with the national average. We compared these coverage differences and rates (indexes) in 2010 and 2018. We then calculated the difference in ART coverage in 2010 and 2018 and the ratio in ART coverage ratios in 2010 and 2018. There are inadequate and incomplete data to compare ART coverages before 2010.

ART coverage difference was calculated by subtracting the ART coverage in one group from another group whereas the ART coverage ratio (index) was calculated by dividing the ART coverage in one group by another group.

*Female: male index* was calculated in two steps. The ART coverage in female and male were calculated, and then a ratio of the ART coverage in female and male was estimated. We then compared the indices over time to check if there was an increasing or decreasing trend. We applied the same steps for ART coverage difference. We used data from the UNAIDS HIV databases on ART coverage between 2000 and 2018.

*Children: adults index* was calculated in two steps. The ART coverage for children and adults were calculated, and then a ratio of the ART coverage for children and adults was estimated. We then compared the indexes for over time to check if there was an increasing or decreasing trend. We applied the same steps for ART coverage difference. We used data from the UNAIDS HIV databases on ART coverage between 2000 and 2018.

*Province: national index* was calculated in three steps. The ART coverage for each region was calculated; the ART coverage for the country was calculated; and the ratio of the ART coverage for each region and ART coverage at national level was estimated. We then compared the indexes for each region over time to check if there was an increasing or decreasing trend. We applied the same steps for ART coverage difference. We used data from Ethiopian national and regional HIV databases on epidemics estimates, ART coverage and from demographic and health survey on HIV testing coverage [11, 12].

## Results

### Evolution of the WHO antiretroviral treatment guidelines

WHO developed the first guidelines on ART for HIV infection in adults and adolescents in 2002 [13]. These supported the launch, by WHO and UNAIDS, of the “3 by 5” initiative in December 2003, with the objective to provide ART to three million PLHIV by the end of 2005. The “3 by 5” initiative was a step towards the goal of making universal access to HIV/AIDS prevention and treatment for all who need them as a human right [14]. At the September 2005 General Assembly High-Level Meeting on HIV/AIDS, United Nations Member States agreed to work towards the goal of “universal access to comprehensive prevention programmes, treatment, care and support” by 2010 [15]. In the June 2006 meeting of the United Nations General Assembly, a resolution was passed towards universal access to HIV/AIDS services [16].

Subsequently, the ART guidelines were comprehensively updated in 2006. The key principles of a public health approach were articulated and expanded in these guidelines: simplified and standardized regimens, including approaches to service delivery, in particular to task shifting, decentralisation, and integration of HIV treatment and care [17, 18]. ART guidelines were further revised to expand the eligibility threshold in 2010 [19]. WHO revised and combined these ART guidelines with other ARV-related guidance into consolidated guidelines in 2013 and 2016 [20, 21].

The guidelines have evolved in response a number of advances in the HIV treatment landscape: new antiretroviral drugs and combinations of them have been developed;^17^ the average cost of first-line therapy has been reduced to less than US$100 per patient per year, from an initial cost of over $10, 000 [22]; point-of-care diagnostics for CD4 cell count, viral load testing, and early infant diagnosis have become available [23]; and the benefit of ARV drugs for HIV prevention has been realised [24, 25]. The implementation of the guidelines benefited from global leadership, strong partnership and advocacy; country adaptation and implementation; a reliable supply of medicines and diagnostics; identification and application of new knowledge at national and global levels [14, 18–21].

Table 1 illustrates the evolution of one of the recommendations (when to start ART with a first-line regimen) of these guidelines. The table shows that the guidelines have evolved from “treating the sickest” to “treating all” [14, 18–21].

**Table 1:** Evolution of the antiretroviral treatment guidelines: when to start first-line treatment

2006	2010	2013	2016
All adolescents and adults with WHO Stage IV HIV disease, irrespective of the CD4 cell count; WHO Stage III disease with consideration of using CD4 cell counts.	All adolescents and adults including pregnant women with HIV infection and CD4 counts of ≤350 cells/mm3, should start ART, regardless of the presence or absence of clinical symptoms. Those with severe or advanced clinical disease (WHO clinical stage 3 or 4) should start ART irrespective of their CD4 cell count. In developing these recommendations, the panel placed high value on avoiding death, disease progression and HIV transmission over and above cost and feasibility.	All adolescents and adults with HIV diagnosis with a CD4 count of 500 cells/mm3 or less, giving priority to initiating ART among those with severe/advanced HIV disease or a CD4 count of 350 cells/mm3 or less. It is also recommended to initiate ART in people with active TB disease and HBV coinfection with severe chronic liver disease, all pregnant and breastfeeding women with HIV, all children younger than five years living with HIV and all individuals with HIV in sero-discordant relationships, regardless of CD4 cell count.	All adults living with HIV, regardless of WHO clinical stage and at any CD4 cell count. Priority will be given to all adults with severe or advanced HIV clinical disease (WHO clinical stage 3 or 4) and adults with CD4 count ≤350 cells/mm3.

In these initiatives, road maps and guidelines, *“access”* has been considered to represent a broad concept, which measures three dimensions: (1) *“availability”* (defined in terms of reachability, affordability and acceptability of services that meet a minimum standard of quality), key services available, affordable and acceptable is an essential precondition for universal access; (2) *“coverage”* (defined as the proportion of a population needing an intervention who receive it); and (3) *“impact”* (defined as reduced new infection rates or as improvements in survival) [26]. We will be focusing on the second and third dimensions of *“access”*, as they not only are higher level indicators (outcome and impact), but also implicitly provide evidence on successes and challenges in the first dimension: there is no *“coverage”* and *“impact”* without *“availability”*.

### Scaling up of antiretroviral treatment: *Coverage*

The number of PLHIV on ART increased from two million in 2005 to more than 23.3 million in 2018; an increase in ART coverage from less than 7% in 2005 to close to 62% in 2018 (Table 2) [27]. By the end of 2018, it was estimated that 79% of PLHIV globally knew their HIV status; among those who knew their HIV status, 78% were accessing ART; and, 86% of people accessing ART had suppressed viral loads [27].

**Table 2:** Scaling up of antiretroviral treatment between 2000 and 2018

Variables	2000	2005	2010	2013	2018
Estimate number of PLHIV (millions): (a)	27.4	30.1	32.4	34.3	37.9
Estimated number of PLHIV on ART (in millions): (b)	0.6	2.0	8.0	13.4	23.3
ART coverage (%): (b/a)X100	2.2	6.6	24.7	39.1	62

Countries are used to reporting the number of people on ART as ever started on ART or currently on ART. However, the databases from UNAIDS and WHO do not provide separate figures on these. Similarly, we were not able to compile and present the data on second-line ART, as it was either not reported by countries or the report was incomplete.

### New HIV infections and deaths: *impact*

**Fig. 1:**
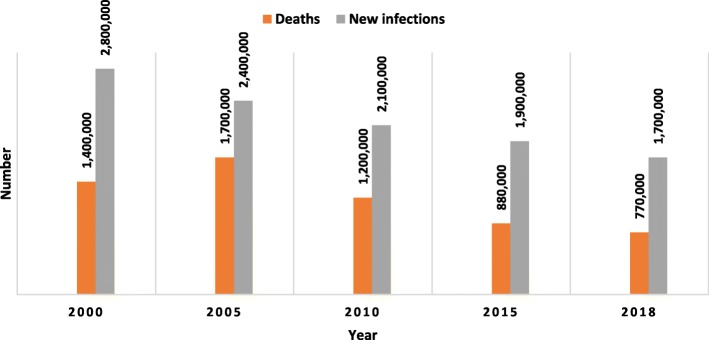
Number of new HIV infections and deaths in people living with HIV, 2000–2018

Expanding the eligibility criteria for ART and using ARV drugs for prevention create opportunities to reduce HIV transmission and save lives. With the scale-up of ART, PLHIV can expect to have a life expectancy similar to that of the general population. Figure 1 shows that the number of new HIV infections dropped from 2.8 million (by 39%) in 2000 and 2.4 million (by 29%) in 2005 to 1.7 million in 2018. On the other hand, the number of deaths dropped by 55% from 1.7 million in 2005 to 0.77 million in 2018 [28].

### Equity in antiretroviral treatment coverage

In spite of the success in increasing ART coverage in all populations and locations, progress varies by region: in eastern and southern Africa, there has been much more gain than other regions; western and central Europe and North America have nearly reached the targets; On the other hand, eastern Europe and central Asia, the Middle East and North Africa and western and central Africa regions are considerably off track. For instance, in eastern and southern Africa, 62% of children were accessing ART while 28% of children were accessing ART in western and central Africa in 2018 [27].

These gaps remain challenges for the ART program at global, regional and national levels. Inequity continues to be a problem and even widened over time (Table 3). This trend has followed the changes in the ART guidelines for treatment initiation. The expanded ART eligibility criteria towards universal ART overlaps with the widened inequity (among population groups (female versus male and adults versus children) and across locations).

**Table 3:** Changes in differences in ART coverage between adults and children and female and male people living with HIV, 2010–2018

Regions	ART coverage															
	2010				2018				2010				2018			
	Adults	Children	Coverage difference	Parity index	Adults	Children	Coverage difference	Parity index	Female	Male	Coverage difference	Parity index	Female	Male	Coverage difference	Parity index
Global	25%	22%	-3%	88%	62%	54%	−8%	87%	25%	25%	0%	100%	68%	55%	−13%	81%
South-east Africa	26%	25%	−1%	96%	67%	62%	−5%	93%	27%	24%	−3%	89%	72%	59%	−13%	82%
West-central Africa	15%	8%	−7%	53%	53%	28%	−25%	53%	16%	13%	−3%	81%	61%	40%	−21%	66%
Middle East and North Africa	10%	11%	1%	110%	32%	35%	3%	109%	10%	10%	0%	100%	35%	31%	−4%	89%
Asia and Pacific	18%	34%	16%	189%	54%	78%	24%	144%	21%	17%	−4%	81%	60%	50%	−10%	83%
Latin America	38%	48%	10%	126%	63%	48%	−15%	76%	36%	35%	−1%	97%	62%	63%	1%	102%
Caribbean	26%	36%	10%	138%	56%	42%	−14%	75%	25%	20%	−5%	80%	62%	50%	−12%	81%
Western and central Europe and North America									67%	68%	1%	101%	77%	78%	1%	101%
Eastern Europe and central Asia									12%	10%	−2%	83%	46%	33%	−13%	72%

Data Source: UNAIDS: http://aidsinfo.unaids.org/

**Fig. 2:**
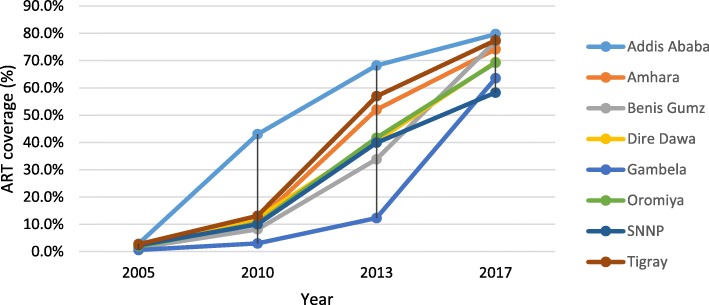
ART coverage at regional level in Ethiopia, 2005, 2010, 2013 and 2017

**Fig. 3:**
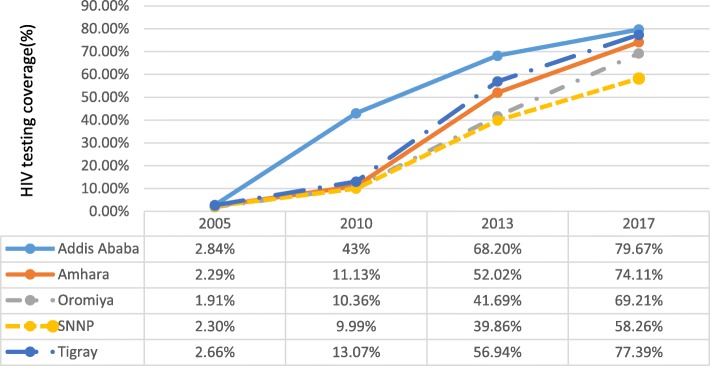
HIV testing coverage at regional level in Ethiopia, 2005, 2011 and 2016

In Ethiopia, ART coverage increased in all regions between 2006 and 2017. ART coverage has converged in all regions, including Gambella region with the highest prevalence of HIV in the country (Fig. 2). The progress in ART coverage in the country and its regions is associated with the increased HIV testing coverage over time. The differences in ART coverage among regions can also be explained by the variability in HIV testing coverage across regions (Figs. 2 and 3). Among regions which contributed more than 90% of the HIV burden in the country, ART coverage is correlated with HIV testing coverage with Pearson correlation coefficient of 0.74.

## Discussion

The WHO’s ART guidelines have evolved over time as new drugs are developed, knowledge and experience in clinical and programmatic management of HIV have improved, the prevention benefit of ARVs has been better recognized and more funding is available. The guidelines have shifted from “treating the sickest” to “treating all” and from “providing ART for clinical benefit” to “providing ART for both clinical and public health benefits”. These treatment guidelines supported and facilitated the proper management and scale-up of ART using a public health approach towards universal access. This has increased ART coverage at global, regional and national levels; and, the number of new HIV infections and deaths among PLHIV dropped markedly. Early lessons learned in scaling up access to treatment fundamentally altered the public health landscape and influenced subsequent actions towards the goal of universal coverage for ART and ending the epidemic [29–32].

Despite the increase in ART coverage at global, regional and national levels, inequity in ART coverage has remained (Tables 2 and 3). What is more worrisome than the existing disparity is the increasing inequity over time in spite of the policies for universal ART coverage. Those populations with better coverage have continued to utilize the services and benefited from the expanded ART initiation guidelines while those which were lagging behind have remained so much more. This phenomenon is also documented as ‘*the inverse care law’*: the availability of good medical care tends to vary inversely with the need for it in the population served [33].

This inequity is happening and increasing in spite of free ART delivery. This is observed mainly in regions with predominant heterosexual HIV epidemic, low HIV testing, and weak health systems capacity much more than others. Parity has been achieved between male and female in regions where there is predominantly male epidemic (Western and Central Europe and North America and Latin America) [28]. However, there is a gap in equity between male and female in ART coverage in regions with predominant heterosexual epidemic (West-central Africa, South-east Africa, and Caribbean) [28]**.**

Differences in HIV testing is one of the reasons that ART coverage among men living with HIV (53%) is less than ART coverage among women (65%) globally. Across different geographic and epidemic settings, men are less likely than women to take an HIV test, which is an entry to treatment [27]. In regions where HIV testing is considerably off track— eastern Europe and central Asia, the Middle East and North Africa and western and central Africa—ART coverage is relatively low. HIV testing coverage remains a particular challenge in western and central Africa, where only an estimated 48% of PLHIV knew their HIV status in 2017. On the other hand, in eastern and southern Africa, there has been continued gains in knowledge of HIV status and linkages to care thanks to a combination of strong domestic leadership and resolute global support. Western and central Europe and North America have also achieved a high testing coverage [27]. These together provide evidence for reinforced action for continuum of care towards increased ART coverage and improved equity.

Weak health systems have undermined efforts to scale up HIV testing and ART in certain regions [27]. Inequity in ART coverage between men and women is more prominent in regions with weak health systems capacity (human resource for health per 1000 population, number of hospital beds per 1000 population and UHC index) than others. The World health statistics 2019 indicates that sub-Saharan Africa and the Caribbean have the weakest health systems among the regions in the globe [34]. We argue that this weak health systems capacity, together with the type of the epidemic and other demand-related factors, can explain the inequity in ART coverage between men and women and children and adults. The data from Ethiopia also show that regions with predominant pastoralist and rural populations (living in regions with weaker health systems capacity) have a lower ART coverage and a huge inequity compared to regions with more urban populations (Figs. 2 and 3) [35, 36].

Countries have been implementing certain interventions that have enabled them to narrow the inequity gap. In Ethiopia, for instance, the framework of primary healthcare, in general, and the public health approach to ART delivery, in particular, includes community engagement and participation, task shifting, decentralization, free provision of HIV services, health systems strengthening [18, 37, 38]. The drop in inequity in ART coverage, mainly among the major regions and regions with huge burden of HIV, can be explained by the improved health systems capacity and the approach that the country has been utilizing to increase primary healthcare (PHC) services [35, 36]. In other countries, community-supported models of care, including task shifting to community health workers, have improved HIV testing and treatment [39–41]. Providing support, including accompanied clinic visits and money for transportation, and removing user fees greatly increases HIV testing and treatment [42–44].

The SDG-3 (ensure healthy lives and promote wellbeing for all at all ages) aims to achieve universal health coverage (UHC) [45]. This requires countries to provide health services to all, without leaving no one behind. Nevertheless, health services utilization is constrained by factors within and outside the health system. In addition, UHC is a supply-side initiative and requires actions that address the demand side of health services utilization. Hence, it is vital that initiatives towards equity in health also consider socio-economic conditions and make sure that the necessary enabling environment is created. This is possible if countries implement the PHC approach (principles and components) that includes multi-sectoral action. A bolder approach, which fully embraces the Alma-Ata vision of PHC, could deliver substantially greater SDG progress, by addressing the wider determinants of health, promoting equity and social justice [45, 46].

In general, there are several lessons that the UHC movement can learn from the successful ART scale up programs, which have been characterized by free service provision and multi-sectoral response: (1) the plan for UHC will be realized only if there is enabling environment for people to utilize services; (2) achieving UHC requires adequacy of not only the supply side but also that of the demand side of health services delivery and utilization; (3) UHC is not a panacea unless it is implemented according to the principles of PHC, public health approach, and overall socio-economic development; and, (4) monitoring UHC should include not only input and process but also impact indicators.

This study has several limitations. Countries used to reporting the number of people on ART as ever started- or currently- on ART; however, the databases from UNAIDS and WHO do not provide separate figures on these. We contend that the progress towards universal HIV treatment requires data on both ever started (cumulative) and currently on ART. Similarly, we were not able to compile and present data on second-line ART, as it was either not reported by countries or the report was incomplete. Nevertheless, this limitation is not systematic that it does not affect the conclusions of this study. As ART programs mature, the need for second-line ART is increasing. Therefore, countries, UNAIDS and WHO should report on both first- and second-line ART. The other limitation is that the study used data aggregated by WHO regions that links together very different countries, which have varied approaches in scaling up ART delivery. Lastly, we have used a vertical program that has been exceptionally well resourced (with an emergency response) anticipating that the lessons learned can be taken to scale to improve PHC in countries most in need of UHC.

## Conclusion

The WHO’s ART guidelines have evolved from “treating the sickest” to “treating all”. This has increased ART coverage, reduced the number of new HIV infections and deaths among PLHIV. In spite of these successes, there has been inequity in ART coverage among population groups (female versus male and adults versus children) and locations. Alarmingly, in some locations and populations, this inequity has widened over time. This gap is much more pronounced in resource-limited settings with weaker health systems capacity, programs with overall lower ART coverage, and predominantly heterosexual HIV epidemic than others. These lessons from the ART program, which suggest that policies towards universality in resource-limited settings may widen the inequity, should warn the global health community that UHC is not a panacea unless countries take precautions and “put the last first”. We argue that PHC and public health approaches, including multi-sectoral actions and community engagement, are vital towards addressing inequity, achieving UHC and leaving no one behind. It is imperative that countries strengthen their PHC systems, as there will not be UHC without strong PHC.

## Data Availability

All supporting data can be accessed from the corresponding author.
